# Non-invasive identification of combined salinity stress and stalk rot disease caused by *Colletotrichum graminicola* in maize using Raman spectroscopy

**DOI:** 10.1038/s41598-023-34937-8

**Published:** 2023-05-11

**Authors:** Samantha Higgins, Ritu Joshi, Isaac Juarez, John S. Bennett, Aidan P. Holman, Michael Kolomiets, Dmitry Kurouski

**Affiliations:** 1 Department of Biochemistry and Biophysics, Texas A&M University, College Station, TX 77843 USA; 2 Department of Toxicology, Texas A&M University, College Station, TX 77843 USA; 3 Department of Plant Pathology and Microbiology, Texas A&M University, College Station, TX 77843 USA; 4 Department of Entomology, Texas A&M University, College Station, TX 77843 USA; 5 Department of Biomedical Engineering, Texas A&M University, College Station, TX 77843 USA

**Keywords:** Plant physiology, Optical physics

## Abstract

Food security is an emerging problem that is faced by our civilization. There are millions of people around the world suffering from various kinds of malnutrition. The number of people that starve will only increase considering the continuous growth of the world’s population. The problem of food security can be addressed by timely detection and identification biotic and abiotic stresses in plants that drastically reduce the crop yield. A growing body of evidence suggests that Raman spectroscopy (RS), an emerging analytical technique, can be used for the confirmatory and non-invasive diagnostics of plant stresses. However, it remains unclear whether RS can efficiently disentangle biotic and abiotic stresses, as well as detect both of them simultaneously in plants. In this work, we modeled a stalk rot disease in corn by inoculating the plant stalks with *Colletotrichum graminicola*. In parallel, we subjected plants to salt stress, as well as challenging plants with both stalk rot disease and salinity stress simultaneously. After the stresses were introduced, Raman spectra were collected from the stalks to reveal stress-specific changes in the plant biochemistry. We found that RS was able to differentiate between stalk rot disease and salinity stresses with 100% accuracy, as well as predict presence of both of those stresses in plants on early and late stages. These results demonstrate that RS is a robust and reliable approach that can be used for confirmatory, non-destructive and label-free diagnostics of biotic and abiotic stresses in plants.

## Introduction

Crop yield is determined by a large number of factors including plant genetics, irrigation and soil conditions. Furthermore, crop yield can be reduced on up to 30% by various plant diseases, such as fungal and viral infections. *Colletotricum graminicola* is a fungus that infects maize stalk, which results in 5–20% corn yield loss in the U.S. alone^1–3^. One can expect that a confirmatory diagnosis of stalk rot can be used to enable site- and dose-specific application of fungicides, which, in turn, will allow for more efficient disease control and maximization of the corn yield^4^. Both PCR and ELISA can be used to detect fungal diseases^5–7^. These molecular techniques are highly specific and sensitive. However, both PCR and ELISA are time and labor consuming. Their costs vary between $20–30 per sample, which limits broad utilization of these molecular methods of analyses by farmers and plant pathologies^5–7^. A growing body of evidence suggests that imaging techniques, such as red–green–blue (RGB), hyperspectral imaging, and thermography can overcome these limitations^8^. Although required equipment for plant imaging is expensive, the direct cost of such imaging analysis is very low^9^. However, imaging techniques suffer from the lack of specificity since identification of the plant disease is primarily based on the change in the leaf color^4^. At the same time, numerous biotic and abiotic factors, such as drought and nitrogen deficiency, can result in the same visual changes in plant texture and coloration as the discussed above biotic stresses^10^. These limitations of molecular and imaging techniques catalyzed the search for alternative technologies that can be used for confirmatory sensing of biotic and abiotic stresses in plants^11–13^.

Our own experimental findings, as well as scientific results reported by other groups show that Raman spectroscopy can be used for confirmatory, non-invasive and non-destructive surveillance of the plant health. In 2018, Farber and Kurouski demonstrated that using RS, four different fungal infections could be detected in maize kernels^13^. Several years later, we showed that citrus greening disease could be diagnosed in both oranges and grapefruits with nearly 100% accuracy^14,15^. Furthermore, it was shown that RS was capable of confirmatory differentiation between citrus greening disease and nutritional deficiency that caused the same yellowness of plant leaves^14–16^. Gupta and co-workers discovered that RS could be used for confirmatory detection of nitrogen deficiency in plants^12^, whereas recently Morey and co-workers found that RS was capable of highly accurate differentiation between the drought and salinity stresses in peanuts^17^. However, it is unclear whether RS could be used to differentiate between biotic and abiotic stresses, as well as identify both of these stresses in the same plant^18,19^.

Recently, Higgins and co-workers demonstrated that RS could be used to differentiate between several biotic and several abiotic stresses in rice^19^. Spectroscopic analysis of plants revealed drastically different intensities of vibrational bands that originated from carotenoids. Higgins and co-workers also performed HPLC analysis of plant carotenoids to demonstrate that observed by RS changes in the concentrations of carotenoids indeed took place in plants. Their results revealed excellent correlation between RS-sensed and HPLC-determined levels of carotenoids in healthy plants, as well as plants infected by both biotic and abiotic stresses^19^.

Expanding upon this, we modeled stalk rot disease in corn by inoculation of corn stalks with *C. graminicola* (group 1)*.* In the second group of plants, we modeled salinity stress by watering plants with 150 mM sodium chloride. Finally, the third group was exposed to both salinity stress and *C. graminicola* infection. We also grew corn at the same experimental conditions that was subjected to neither one of those stresses (control). Next, we collected Raman spectra from stalks of all four groups of plants on day 2, 4, 6, and 8.

## Materials and methods

### Plant and fungal materials

Seeds of B73 maize genotype were planted with 4–6 seeds per pot in Metro Mix 360 RSi soil (Sun Gro Horticulture). After germination, maize seedlings were thinned to one plant per pot within the next two weeks. Maize was watered every 3–4 days and 20 g of Osmocote Blend 19-5-9 slow-release fertilizer (Everris NA Inc.) were applied to each pot at about two and six weeks after planting. *C. graminicola* (1.001 strain) was cultured from stock plates from the lab of Dr. Young-Ki Jo (Texas A&M University) on full strength potato dextrose agar for at least 14 days at 23–25 °C. Spore suspensions were prepared as previously described^20^. Inoculations were performed at the time point on which 50% of the plants silked (mid-silking). The bottom four internodes above the last node with brace roots were wounded with an 18G hypodermic needle inserted to 1/4 inch depth. Sterile cotton swabs were dipped in spore suspension of 1 × 10^6^ spores/mL of *C. graminicola* and wrapped in place on the wound site with parafilm to create a humid chamber. Infections were allowed to progress to selected time points of 2, 4, 6 and 8 dpi. In total, 10 plants were analyzed per treatment group. In parallel, 10 B73 plants were subjected to watering with 150 mM sodium chloride to induce salinity stress. Finally, 10 plants were subjected to the salinity stress and inoculated with *C. graminicola*, as discussed above, while 10 untreated plants served as the control group.

### Raman spectroscopy

Raman spectra were collected from the surface of 2nd and 4th internodes with a hand-held Resolve Agilent spectrometer equipped with an 830-nm laser source. The following experimental parameters were used for all collected spectra: 1 s integration time, 495 mW power, and baseline spectral subtraction by device software. Fifty spectra were collected from each group of plants. Spectra shown in the manuscript are raw baseline corrected, without smoothing.

### Spectral data analysis

Data preprocessing is an important preliminary step for the data analysis. Primarily because during the acquisition of spectra, the acquired spectra are influenced by multiple sources of signal noise such as sample background and instrument performance which can lead to deterioration of the spectral data quality^21^. Preprocessing of spectra aims to eliminate or minimize the aforementioned impacts to enhance the multivariate regression, classification model, or exploratory analysis that will follow^22^. In this study, standard normal variate (SNV) was used as a preprocessing method for spectral analysis. SNV is normally considered as a scatter correction method that is designed to reduce the (physical) variability between samples caused by scatter or used to adjust for baseline shifts between samples^22^. The basic format of SNV is given below:

1 $$\frac{{\mathrm{x}}_{\mathrm{org}}- {\mathrm{a}}_{0}}{{\mathrm{a}}_{1}}.$$

Here, a_0_ is the average value of the sample spectrum to be corrected, and a_1_ is the standard deviation of the sample spectrum. Data preprocessing represents the process of cleaning and preparing the data for classification or for prediction purposes.

### Machine learning model

In this work, we utilized partial least-squared discriminant analysis (PLS-DA). This supervised version of principal component analysis achieves dimensionality reduction with the information of target variables together with the good insight into the causes of discrimination through weights and loadings that assists in conducting exploratory data analysis^23^. The PLS-DA analysis was performed with a total of 800 samples (Table 1) which were divided into two groups: calibration set and validation set. All calculations and data analyses were carried out using MATLAB 2022b (MathWorks, Inc., Natick, MA, USA).

**Table 1:** Summary of the data used for PLS-DA analysis.

Days	Number of samples			
	Control	Fungus	NaCl	Fungus + NaCl
2	50	50	50	50
4	50	50	50	50
6	50	50	50	50
8	50	50	50	50

### Permissions or licenses

The authors have a permission to grow plants and collect plant materials according to the policy of Texas A&M University. All methods were performed on plants, including the collection of plant material, comply with institutional, national, and international guidelines and legislation.

## Results and discussion

**Figure 1:**
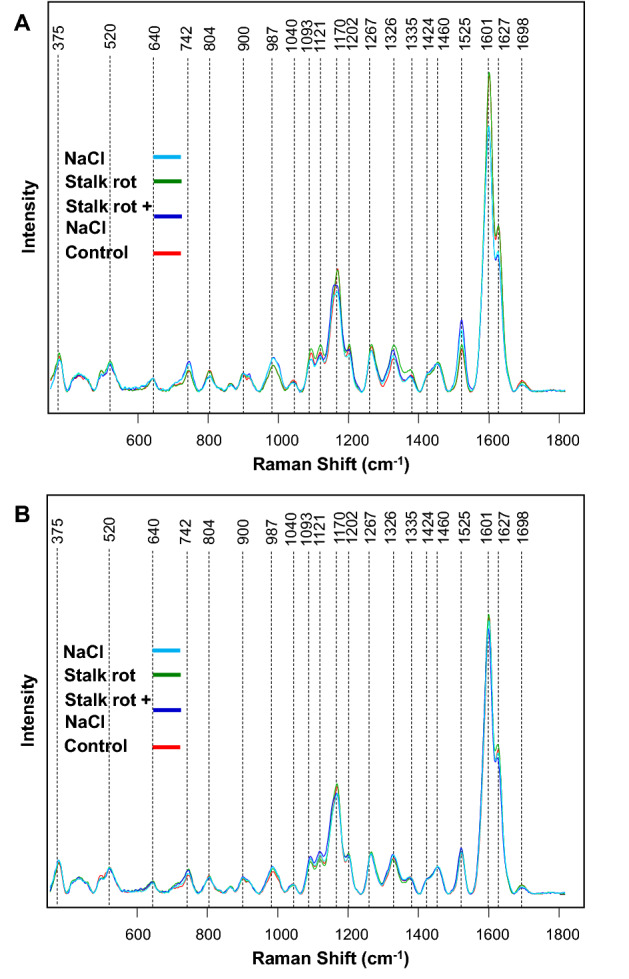
Raman spectra acquired from the stalks of corn plants exposed to salinity stress (NaCl), stalk rot disease (stalk rot), both salinity and stalk rot disease (Stalk rot + NaCl), as well as control plants (control) at day 2 (**A**) and day 8 (**B**).

In the Raman spectra acquired from the stalks of healthy corn, we observed rational bands that can be assigned to phenylpropanoids (1601–1627 cm^−1^) and which dominate the spectra. We also observed vibrational bands that can be assigned to pectin (742 cm^−1^), cellulose (520, 915, 1040, 1093 and 1121 cm^−1^), carotenoids (1525 cm^−1^), carboxylic acids (1698 cm^−1^), and aliphatic vibrations (1326, 1335, 1424, and 1460 cm^−1^) (Fig. 1A and Table 2).

**Table 2:** Vibrational band assignments for wheat leaf spectra.

Band	Vibrational mode	Assignment
375	Associated with cellulose crystallinity	Cellulose^24^
520	ν(C–O–C) Glycosidic	Cellulose^25^
640	*δ*(C–C)	Lignin^26^
742	γ(C–O–H) of COOH	Pectin^27^
804	*δ* ring vibration	Terpenes^28^
900	ν(C–O–C) In plane, symmetric	Cellulose, lignin^25^
987	*ν*(CO)_ring_, *ν*(CC)_ring_, β(CCH)	Carbohydrates^29^
1040	ν(C–O) + ν(C–C) + δ(C–O–H)	Cellulose, lignin^25^
1093	ν(C–O) + ν(C–C) + δ(C–O–H)	Carbohydrates^30^
1121	ν(C–O) + ν(C–C) + δ(C–O–H)	Carbohydrates^30^
1170	C–OH	Lignin^31^
1202	Aromatic ring modes of phenylalanine and tyrosine	Proteins^32^
1267	Guaiacyl ring breathing, C-O stretching (aromatic); –C = C–	Lignin^33^, unsaturated fatty acids^34^
1326	δCH_2_ bending	Aliphatics, cellulose, lignin^25^
1335	δ(CH_2_) + δ(CH_3_)	Aliphatics^35^
1424–1460	δ(CH_2_) + δ(CH_3_)	Aliphatics^35^
1525	–C = C– (in plane)	Carotenoids^36,37^
1601–1627	ν(C–C) Aromatic ring + σ(CH)	Lignin^38,39^
1698	COOH	Carboxylic acids

Similar vibrational bands were observed in the Raman spectra acquired at day 2 from stalks of corn that were infected by *C. graminicola*, Fig. 1A. At the same time, we observed a small decrease in the intensities of most of vibrational bands compared to those observed in the Raman spectra of healthy corn. These results show that fungal infection causes substantial changes in the plant biochemistry, which are sensed by RS. These results are in a good agreement with the previously reported results by Farber and co-workers^40^. Even greater magnitude of changes in the discussed above vibrational bands was observed for the spectra acquired from corn subjected to salinity stress and combined salinity and stalk rot disease. Specifically, we observed a drastic decrease in the intensity of phenylpropanoids, cellulose and lignin vibrations. We also found that intensity of pectin and carbohydrate bands increased in the spectra acquired from plants exposed to salinity stress and combined salinity and stalk rot disease compared to those observed in healthy plants and corn infected by *C. graminicola*. These results point to the drastically different response of corn to the discussed above biotic (*C. graminicola*) and salinity stresses. Spectroscopic analysis of the same plants at day 8 after the initiation of the stresses revealed similar changes in the intensities of the vibrational bands compared to those observed at day 2, Fig. 1B. These results suggest that changes in plant biochemistry that were induced by these stresses persist overtime.

Next, we used PLS-DA, a chemometric method that allows for a quantitative assessment of the discussed above differences in the acquired Raman spectra. Specifically, PLS-DA model combined with SNV preprocessing was able to demonstrate 100% accurate differentiation between the control, salinity and *C. graminicola* stresses, as well as between the plants that were exposed to *C. graminicola* and salinity stresses together at day 2, 4, 6 and 8, Table 3, Fig. S1 and Table S1.

**Table 3:** Results of PLS-DA analysis results for control and stress groups.

Stress group accuracy	Control	Fungus	NaCl	Fungus + NaCl
Day 2	100%	100%	100%	100%
Day 4	100%	100%	100%	100%
Day 6	100%	100%	100%	100%
Day 8	100%	100%	100%	100%

In the previously study reported by our group, Farber and co-workers demonstrated that RS could be used to determine the extent to which different corn varieties were resistant to *C. graminicola*^40^. To demonstrate this, we correlated the size of lesions with the changes in the intensity of vibrational bands that could be assigned to carotenoids, phenylpropanoids and cellulose. We found that different varieties of corn, specifically B73, lox4-7 and MP305, exhibited slightly different spectroscopic changes after infection with *C. graminicola*^40^. These results demonstrated that RS could be used for selection procedures for genetic improvement programs.

## Conclusions

Our results show that RS is capable of 100% accurate differentiation between biotic (stalk rot disease) and abiotic stresses (salinity) in the same crop. Furthermore, RS can be used to detect presence of both of these stresses in corn and differentiate such combined biotic-abiotic stress from individual biotic and abiotic stresses. One may wonder whether RS could be used to differentiate between different biotic stresses. Recently reported results by Higgins and co-workers showed that RS could be used to differentiate between aphid stress and viral disease in wheat^18^. The researchers also demonstrated that RS could disentangle between abiotic stresses, such as drought and nitrogen deficiency. The reported by Higgins and co-workers results of HPLC-based analysis of carotenoid profiles of wheat leaves from all those group of plants demonstrated that Raman-based identification of different biotic and abiotic stresses is based on the sensing of chemical profile of carotenoids, which are unique for different biotic and abiotic stresses^18^. Although the current study is critically focused on elucidation of the feasibility of Raman-based differentiation between drought and stalk rot disease, as well as diagnostics of the dual stress in corn, the reported by Higgins and co-workers result suggest that RS could be used for differentiation between different biotic and abiotic stresses in corn.

## Supplementary Information

Supplementary Information.

## Data Availability

The data that support the findings of this study are available from Dr. Kurouski but restrictions apply to the availability of these data, which were used under license for the current study, and so are not publicly available. Data are however available from the authors upon reasonable request and with permission of Dr. Kurouski.
