# Supplementary material for: Non-invasive identification of combined salinity stress and stalk rot disease caused by Colletotrichum graminicola in maize using Raman spectroscopy
Source: Sci Rep. 2023 May 11;13:7661. doi: 10.1038/s41598-023-34937-8 (PMC10175297; doi:10.1038/s41598-023-34937-8)
Supplement: Supplementary file 1 — Supplementary Information. [file 41598_2023_34937_MOESM1_ESM.docx]

Non-Invasive Identification of Combined Salinity Stress and Stalk Rot Disease Caused by *Colletotrichum graminicola* in Maize Using Raman Spectroscopy

Samantha Higgins^1†^, Ritu Joshi^1†^, Isaac Juarez,^2^ John S. Bennett,^3^ Aidan P. Holman^4^, Michael Kolomiets^3*^ and Dmitry Kurouski^1,2,5*^

† Authors contributed equally to this work.

Department of Biochemistry and Biophysics, Texas A&M University, College Station, TX 77843, United States

Department of Toxicology, Texas A&M University, College Station, TX 77843, United States

1. Department of Plant Pathology and Microbiology, Texas A&M University, College Station, TX, 77843, United States
2. Department of Entomology, Texas A&M University, College Station, TX, 77843, United States
3. Department of Biomedical Engineering, Texas A&M University, College Station, TX, 77843, United States

Supporting Information


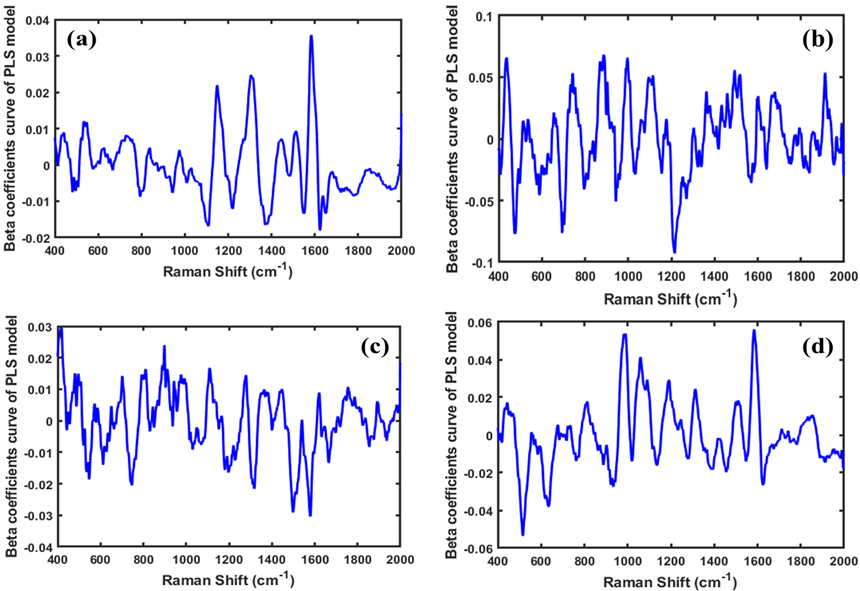


Figure S1. PLS-DA beta coefficient plots for day 2 (a), day 4 (b), day 6 (c), and day 8 (d) for corn plants. The beta coefficient is the degree of change in the outcome variable for every 1-unit of change in the predictor variable. Here, beta-coefficient plots show the spectral variations between various groups of samples. The beta plot is an essential for the localization of wavenumbers in multivariate analysis since it helps reveal information about the chemical characteristics of compounds. Certain important peaks were observed in the beta coefficient plot for day 2 (a), day 4 (b), day 6 (c), and day 8 (d), respectively. These are the regions which display the spectral differences between various groups of samples, shows similar peaks in the spectral region sensitive to control, fungus, NaCl, and fungal + NaCl. Thus, the beta-coefficient obtained from the PLS-DA method is attributable to the variation in control samples when compare with fungus, NaCl, and fungus + NaCl samples.

Table S1. Raman spectroscopy-based PLS-DA classification results for control and stress groups on days 2, 4, 6, and 8.


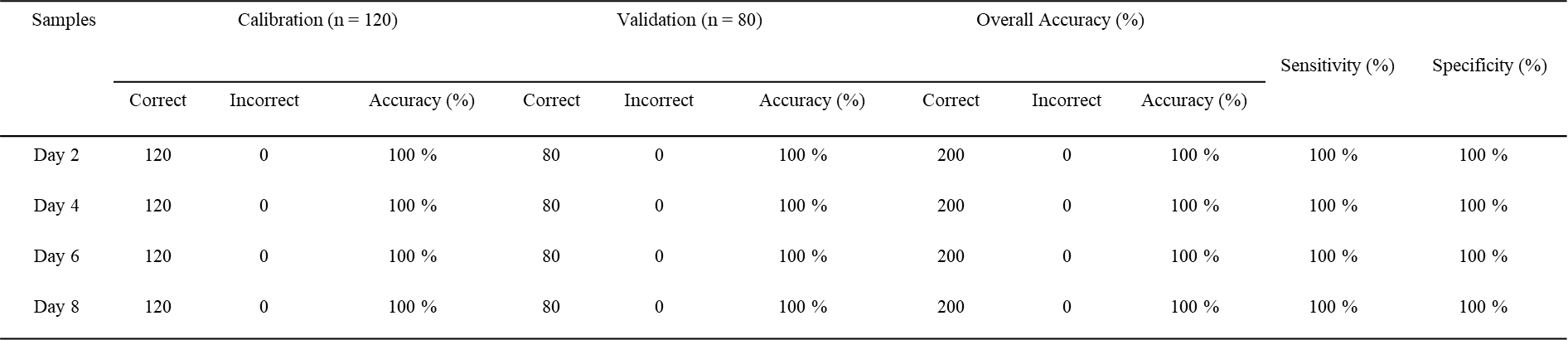


The sensitivity evaluates how effectively the model can accurately categorize samples from the case class, whereas the specificity represents how well the model can predict samples from the control class. The parameters for quality analysis of the samples were calculated based on the calculated response by the model as a true positive (a positive response for a positive sample), false positive (a positive response for a negative sample), true negative (a negative response for a negative sample), and false negative (a negative response for a positive sample).

Sensitivity = (No. of detected pure samples/No. of total pure samples) ∗ 100;

Specificity = (No. of detected adulterated samples/No. of total adulterated samples) ∗ 100;
